# Durable and Robust Janus Membranes with Asymmetric Wettability Based on Poly (Vinylidene Fluoride)/Polyvinyl Alcohol for Oil–Water Separation

**DOI:** 10.3390/ma19020363

**Published:** 2026-01-16

**Authors:** Yawen Chang, Ruihong Sun, Fujuan Liu

**Affiliations:** National Engineering Laboratory for Modern Silk, College of Textile and Clothing Engineering, Soochow University, 199 Ren-Ai Road, Suzhou 215123, China; 20225215004@alu.suda.edu.cn (Y.C.); 20245215009@stu.suda.edu.cn (R.S.)

**Keywords:** oil–water separation materials, Janus fiber membranes, separation efficiency, layer-by-layer electrospinning, mechanical properties

## Abstract

With the acceleration of industrialization, the problems of water resource pollution and shortage caused by oil spills and industrial wastewater discharge have become increasingly severe, posing a major threat to ecological sustainable development. Therefore, efficient oil–water separation technology has become a key breakthrough to alleviate this crisis. In this study, Janus membranes with asymmetric wettability were prepared by layer-by-layer electrospinning. The influence of the thickness ratio between the hydrophobic layer and the hydrophilic layer on the mechanical properties, separation flux, and oil–water mixture efficiency of the Janus membranes was examined, and an optimized membrane configuration was determined: the optimal thickness ratio between hydrophobic and hydrophilic layers was 4:6. Under these conditions, the fracture stress of the fiber membranes reached 99% MPa, the fracture strain was 55.63 ± 4.77%, the separation flux values were 1888.22 and 1042.66 L m^−2^ h^−1^ for the oil–water mixture and water-in-oil emulsion, respectively, with the separation efficiencies all exceeding 99%. After 50 cycles of separation for two different oil-in-water emulsions, the separation flux and separation efficiency of the optimal sample remained relatively stable, demonstrating strong practicability. In general, the Janus fiber membranes met the expected requirements, laying a good foundation for future applications in oil–water separation, floating oil collection in water, and other fields.

## 1. Introduction

In ancient Roman mythology, Janus was the god symbolizing beginnings and endings, also known as the two-faced god [1]. Janus membranes refer to a type of membrane with opposite physical or chemical properties on both sides. The bilateral asymmetry is generally manifested as differences in morphology, wettability, charge, pore size distribution, etc. [2,3,4]. Therefore, the Janus fiber membrane with asymmetric wettability represents the fact that the wettability of the front and back sides of the fiber membrane is completely opposite, compared to the liquid. In an air environment, the wettability of both sides of the fiber membrane can be hydrophobic–hydrophilic or lipophilic–oleophobic. Compared with the single-wettability membranes, the special structure of the Janus fiber membranes with asymmetric wettability provides a self-driven force, enabling the directional transportation of specific liquids [5,6].

This property significantly enhances gas–liquid transport efficiency, driving rapid advancements in traditional and emerging fields such as seawater desalination [7], unidirectional liquid transportation [8,9], wound dressings [10], mist collection [11], emulsification [12,13], and oil–water separation [14,15,16]. In biomedicine, the concept of unidirectional fluid transport can be applied to the management of biological fluids (e.g., blood, exudate), meaning that Janus membranes with asymmetric wettability are usually used as wound dressings for sterilization, disinfection, hemostasis, etc. [17]. Hu et al. [18] successfully prepared a multifunctional polycaprolactone/chitosan Janus membrane (PCJM) with asymmetric wettability: the hydrophobic layer, composed of dense and disordered polycaprolactone, prevents bacterial adhesion, resists scaling, and improves mechanical properties; the hydrophilic layer, a porous chitosan sponge, facilitates the cells and proteins adhesion and blood absorption. In addition, PCJM exhibited excellent biocompatibility, significantly shortened the coagulation time, effectively promoted the gingival healing of Beagle tooth extraction wounds, and showed great potential for clinical transformation and large-scale production. The wetting characteristics of the Janus fiber membranes with asymmetric wettability endow them with the liquid screening function [19], highlighting their application potential in oil–water separation. Wang et al. [20] prepared an intelligent dual-functional Janus mesh membrane, which integrated selective directional oil–water delivery for efficient on-demand emulsion separation, using a combined strategy of single-sided oxidation, hydrophobic treatment, and single-sided spraying. Inspired by the bionic strategy (lotus leaf and mussel), Yu et al. [21] fabricated a Janus polydopamine–polytetrafluoroethylene/titanium dioxide membrane via centrifugal spinning technology. This membrane achieved >99% separation efficiency for both water–oil mixtures and emulsions, remained >98.5% efficient after 24 h in the corrosive solution, and exhibited excellent reusability, acid and alkali resistance, and mechanical stability.

Typically, the preparation methods for Janus fiber membranes with asymmetric wettability are mainly categorized into two types: asymmetric modification and asymmetric manufacturing. Asymmetric modification involves single- or double-sided modification based on fiber membrane substrates, with surface modification achievable through methods such as spraying, plasma treatment, vapor deposition, and photo catalysis [19,22,23,24]. Usually, the fiber membranes that undergo double-sided modification are obtained through continuous single-sided modification. That is, after completing the modification of one side, the fiber membranes are turned over and further modified to produce Janus fiber membranes with asymmetric wettability [25]. However, asymmetric modification imposes stringent requirements for the modified materials and modification conditions. Controlling the scope and degree of modification is challenging, and there is a risk of the modified layer falling off [26]. Yu et al. [27] proposed a simple, effective and low-cost method for preparing Janus polydimethylsiloxane (PDMS) membranes. Using hydrophobic PDMS membranes as the substrate, after perforating them, the single-sided PDMS membranes were grafted with hydrophilic polyethylene glycol (PEG) molecules induced by air plasma to form Janus membranes with super-wettability. In addition, the Janus membrane can still maintain its unidirectional water delivery capacity after 15 cycles and exhibits excellent stretchability and biocompatibility.

In contrast, asymmetric manufacturing generally refers to separately preparing two or more layers of fiber membranes and combining them through specific techniques to form Janus fiber membranes. The induced phase-separation preparation process is relatively complex. Commonly employed asymmetric manufacturing methods with simpler operation procedures include layer-by-layer electrospinning [28] and vacuum filtration deposition [29,30,31]. Among these, layer-by-layer electrospinning is widely utilized for the preparing asymmetrically wetted Janus fiber membranes, owing to its advantages such as the simple preparation process and low energy consumption [32]. Based on this technology, research on Janus membranes has been expanded across multiple polymer systems [33,34,35]. For instance, polyacrylonitrile (PAN) acts as a functional matrix with excellent spinnability and mechanical strength [36]. Polyvinyl alcohol (PVA), endowed with high hydrophilicity and easy crosslinking capacity, is used to fabricate efficient hydrophilic layers [37]. Polystyrene (PS), in turn, serves as a template for constructing tailored micro- and nanostructures [38]. Zhou et al. [35] fabricated a porous hydrophilic layer using hydrophilic PAN and polyvinylpyrrolidone (PVP), and hydrophobic layers using hydrophobic polystyrene (PCL). Porous Janus nanofiber membranes were successfully obtained by continuous electrospinning technology combined with post-treatment technology. Due to its unique porous structure and bilateral anisotropy, these Janus nanofiber membranes simultaneously achieved both high filtration efficiency (99.98%) and low air resistance (134.7 Pa), demonstrating the great potential of this type of fiber membrane in personal protection applications.

Although layered electrospinning has been successfully applied to prepare various high-performance Janus separation membranes, existing research predominantly focuses on developing novel composite systems or optimizing individual separation metrics (such as flux and efficiency). A critical yet overlooked scientific gap persists: the systematic influence and synergistic regulation of core structural parameters, especially the impact of the thickness ratio of the hydrophobic/hydrophilic layer on the overall performance of the composite membrane, particularly when shifting research focus from standalone functional layer preparation/optimization to integrating distinct layers into complete Janus systems.

Accordingly, this study aims to focus on Janus membrane structural design. Based on our previously developed hydrophobic (PVDF/SiO_2_) [39] and hydrophilic (cPVA/PEG) [40] layers, we systematically modulated their thickness ratio to investigate the influence of this key structural variable on the comprehensive performance of composite Janus membranes. Furthermore, the comprehensive performance characterization of optimized samples were conducted, covering surface wettability, long-term cycling separation stability, and flexibility. Therefore, these efforts aim to clarify how to achieve simultaneous optimization of the mechanical stability, separation efficiency, and operational durability of Janus membranes by precisely regulating structural parameters (thickness ratio) under the premise of fixed material properties.

## 2. Experimental

### 2.1. Materials

Poly (vinylidene fluoride) (PVDF, Mw = 500,000 g/mol) was obtained from Shanghai D&B Biological Science and Technology Co., Ltd. (Shanghai, China). Hydrophobic nanofumed silica (SiO_2_, Mw = 60.08 g/mol, 7–40 nm) was supplied by Shanghai Maclin Biochemical Technology Co., Ltd. (Shanghai, China). Polyvinyl alcohol 1788 (PVA1788, Mw = 85,000 g/mol) and Glutaraldehyde (GA, 50% in H_2_O) were provided by Shanghai Aladdin Biochemical Technology Co., Ltd. (Shanghai, China). Polyethylene glycol 4000 (PEG4000, Mw = 4000 g/mol) and Span80 (Mw = 428.61 g/mol) were purchased from Shanghai Yuanye Bio-Technology Co., Ltd. (Shanghai, China). N, N-dimethyformamide (DMF), dichloromethane (DCM), n-hexane and Anhydrous ethanol were all obtained from Jiangsu Qiangsheng Functional Chemistry Co., Ltd. (Suzhou, China). Water-based dyeing agent Alcian Blue (AB, Mw = 1298.88 g/mol) was obtained from Sinopharm Chemical Reagent Co., Ltd. (Shanghai, China). Oil-based dyeing agent Sudan II (Mw = 276.33 g/mol) was produced by Shanghai Adamasi Reagents Co., Ltd. (Shanghai, China). All reagents are analytically pure.

### 2.2. Preparation of Asymmetric Wettability Janus Composite Nanofiber Membranes

Electrospinning solutions were obtained by successively dissolving PVDF and SiO_2_ nanoparticles in DMF solvent with stirring for 12 h at room temperature. The SiO_2_ concentration accounted for 1 wt.% of the total solution, and the PVDF concentration was maintained at 12 wt.% [39]. Meanwhile, a certain amount of PVA and PEG powders were dissolved in deionized water and stirred in a water bath with a constant temperature of 70 °C for 6 h to prepare a 10 wt.% spinning solution, in which the mass ratio of PVA to PEG was 9:1. The solution was then kept at room temperature for 3 h to remove any entrapped bubbles. The obtained PVA/PEG spinning solution was mixed with the GA solution at a specific volume ratio (85/15) under stirring, resulting in the formation of cross-linked PVA/PEG spinning solutions [40].

**Figure 1:**
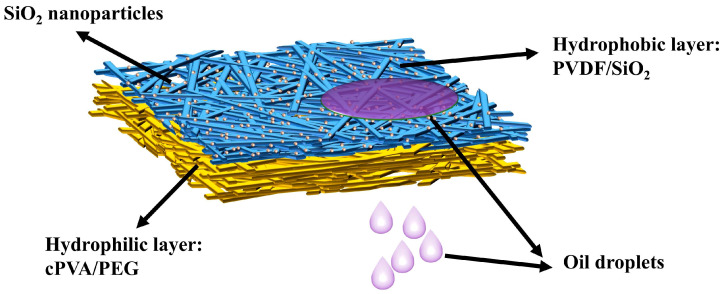
The structure of the composite Janus nanofiber membrane.

All the fabricated solutions were loaded into syringes for electrospinning. The applied voltages of the PVDF/SiO_2_ and cPVA/PEG solutions were set at 24 kV and 20 kV, respectively, while other consistent process parameters included the needle-to-drum receiving device distance of 12 cm, the spinning speed of 1 mL/h, and the drum rotation speed of 400 rpm. In addition, the indoor temperature and relative humidity (RH) were maintained at 22 °C and 50%, respectively, during the preparation process. Janus composite nanofiber membranes with asymmetric wettability were fabricated by layer-by-layer electrospinning, and a diagram showing their specific structure is illustrated in Figure 1. Post-spinning, the as-obtained fiber membranes were dried in an oven at 60 °C for 3 h to completely remove the residual solvent.

In the subsequent experiments, precise control over the fiber membrane thickness can be achieved by adjusting the spinning time. Among them, the thickness of the fiber membranes was measured using a digital micrometer. The total thickness of the fiber membranes was controlled at 50 ± 5 μm, while the thickness ratios of the hydrophobic and hydrophilic layers were systematically varied to 8:2, 6:4, 5:5, 4:6, and 2:8.

### 2.3. Preparation of Oil–Water Mixtures and Emulsions for Separation Tests

DCM and deionized water were stained with Sudan II and AB, respectively. An oil–water mixed solution with a DCM to deionized water volume ratio of 1:1 was prepared. For the water-in-oil emulsion preparation, 1 g of water was mixed with 99 g of oil (DCM, n-hexane), followed by adding 0.1 g of Span80. The mixture was stirred with a magnetic stirrer for 2 h and then ultra-sonicated in an ultrasonic cleaner for 2 h to obtain a surfactant-stabilized emulsion. This emulsification step aims to establish a uniform and stable model system during separation tests, thereby ensuring the reliability and reproducibility of separation performance evaluations.

### 2.4. Characterization

The tensile properties were characterized via a universal testing machine (Instron 5967, Instron, Norwood, MA, USA). The variations in underoil water contact angle and underwater oil contact angle on the surface of the Janus fiber membrane were also recorded using a contact angle tester (OCA40, Dataphysics, Filderstadt, Germany). The separation performance of the Janus composite nanofiber membrane was quantitatively evaluated by the separation flux and separation efficiency. The calculation method for the separation flux (F) of the fiber membrane is presented in (Equation (1)): [41]

(1) $$F\;\left(Lm^{-2}h^{-1}\right)=\frac{V}{s\;\times \;\Delta T}\;$$

where V is the volume (L) of the filtered liquid, s represents the effective separation area (m^2^) of the membrane, and ΔT denotes the effective separation time (h) of the oil–water separation membrane.

The formula of estimating the separation efficiency (E) of the fiber membrane is shown in (Equation (2)): [42]

(2) $$E\;\left(\%\right)=\frac{m}{m_{0}}\;\times \;100\%$$

where m represents the mass of the oil obtained after separation (g), and $m_{0}$ is the mass of the oil in the solution before separation (g).

### 2.5. Oil–Water Separation Performance Test

**Figure 2:**
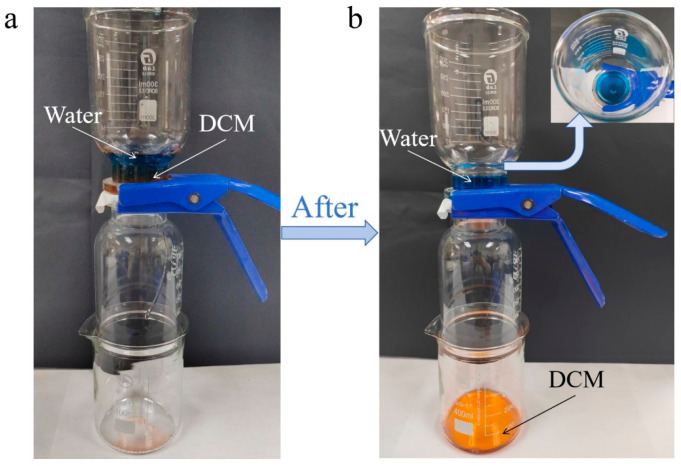
Separation process of oil–water mixtures using Janus composite nanofiber membranes: (**a**) initial state of the separation: the oil-water mixture is placed above the membrane (hydrophobic side up); (**b**) state after separation: the water phase is blocked, while the oil phase (DCM) passes through the membrane, achieving separation.

An oil–water separation device self-developed in the laboratory was adopted to evaluate the separation performance under gravitational action. Figure 2 demonstrates the process of separating the oil–water mixture using Janus composite nanofiber membranes. As illustrated in Figure 2a, the fabricated Janus composite nanofiber membrane was fixed between two filter cups, with its hydrophobic side facing upward. The oil–water mixture (or water-in-oil emulsion) was then poured into the upper cup. After a period of time (as shown in Figure 2b), due to the hydrophobic nature of the corresponding layer, water was blocked and could not permeate the Janus fiber membrane, while the oil phase (e.g., DCM) passed through completely and was collected in the lower vessel, thereby achieving separation. The entire separation process was driven solely by gravity. The separation flux (F) and separation efficiency (E) were calculated according to Equations (1) and (2), respectively, presented in Section 2.4.

## 3. Results and Discussion

### 3.1. Tensile Properties

**Figure 3:**
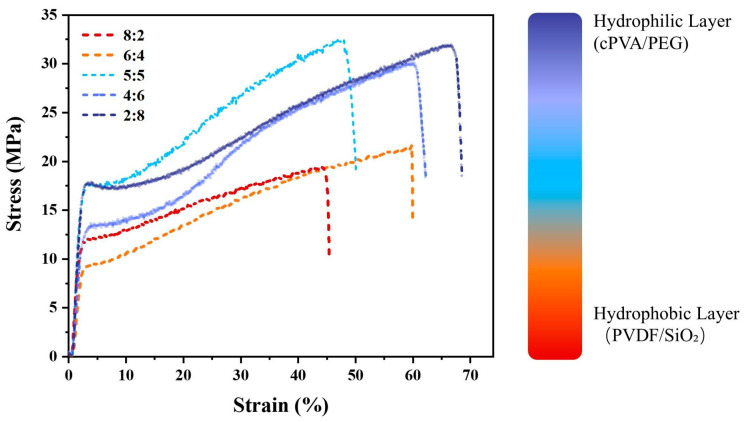
Tensile properties of Janus composite nanofiber membranes with varying thickness ratios of hydrophobic and hydrophilic layers (8:2, 6:4, 5:5, 4:6 and 2:8, respectively).

Given the significant disparity in the mechanical properties of the hydrophobic and hydrophilic layers of the Janus composite nanofiber membrane, it is necessary to test whether the layer-by-layer electrospinning method can maintain the excellent mechanical properties of the Janus fiber membrane. The mechanical properties of Janus composite nanofiber membranes with varying thickness ratios of hydrophobic and hydrophilic layers were analyzed. Figure 3 illustrates the stress–strain curves, and Table 1 presents the specific data for breaking stress and breaking strain. As the thickness of the hydrophilic layer increased, the breaking stress of the composite fiber membrane showed a trend of increasing first and then decreasing. When the thickness ratio of the hydrophobic layer to the hydrophilic layer was 8:2, the breaking stress of the membrane reached the minimum value (20.95 MPa). When the ratio changed to 6:4, the breaking stress increased slightly by 0.07 MPa. The highest breaking stress (32.70 MPa) was observed while the hydrophilic and hydrophobic layers had equal the thicknesses. Since then, although a slight decrease in breaking stress as the hydrophilic layer thickness continued to increase, it remained above 30 MPa. Meanwhile, the breaking strain of the composite fiber membrane exhibited a trend of increasing, decreasing, and then increasing again with the growth of the hydrophilic layer thickness. At a thickness ratio of 8:2, the breaking strain was the lowest at 42.48%. When the ratio became 6:4, the breaking strain significantly increased to 61.81%. With the continuous increase in the hydrophilic layer thickness, the breaking strain first dropped substantially to 48.85% and then steadily rose to 70.35%. In conclusion, the mechanical properties of the composite fiber membrane were generally improved with the increase in the hydrophilic layer thickness.

### 3.2. Separation Performance of Oil–Water Mixture

**Figure 4:**
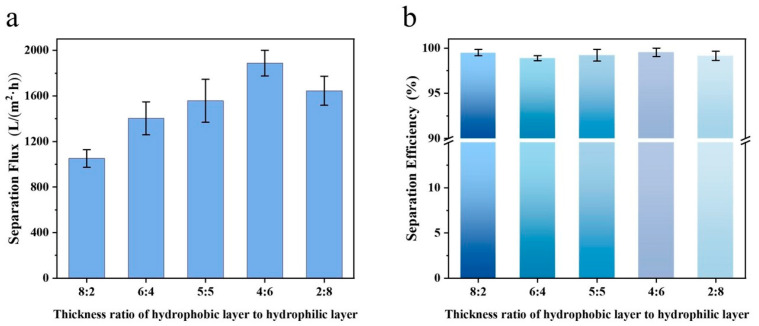
The separation performance of Janus composite nanofiber membranes for oil–water mixtures under different thickness ratios of hydrophobic and hydrophilic layers: (**a**) separation flux; (**b**) separation efficiency.

Following the test method described in Section 2.5, the separation performance of Janus membranes with varying hydrophobic-to-hydrophilic layer thickness ratios was evaluated for oil–water mixtures (Figure 4). It can be seen from Figure 4a that as the thickness of the hydrophilic layer in the composite fiber membrane gradually increased, the separation flux of the fiber membrane exhibited a trend of first increasing and then decreasing. When the thickness ratio was 4:6, the separation flux reached the maximum value (1888.22 L m^−2^ h^−1^). This maximum is attributed to a synergistic balance: the hydrophobic layer provides a sufficient and rapid transport pathway for the oil phase, whereas the hydrophilic layer effectively blocks water penetration without imposing excessive flow resistance. When the ratio was further reduced to 2:8, the separation flux of the fiber membrane decreased slightly, which may be attributed to the excessively thin hydrophobic layer that impaired the directional transportation of the oil. As can be seen from Figure 4b, the values for the separation efficiency of the composite fiber membrane for the oil–water mixture were all above 98%. Specifically, when the thickness ratio of the hydrophobic layer to the hydrophilic layer was 4:6, the separation efficiency of the fiber membrane was the highest, reaching 99.53%. The nearly complete separation efficiency under all conditions confirms the efficacy of the asymmetric wettability design inherent to the Janus structure, which ensures selective permeation of oil or water regardless of the specific layer thickness.

### 3.3. Separation Performance of Water-in-Oil Emulsion

**Figure 5:**
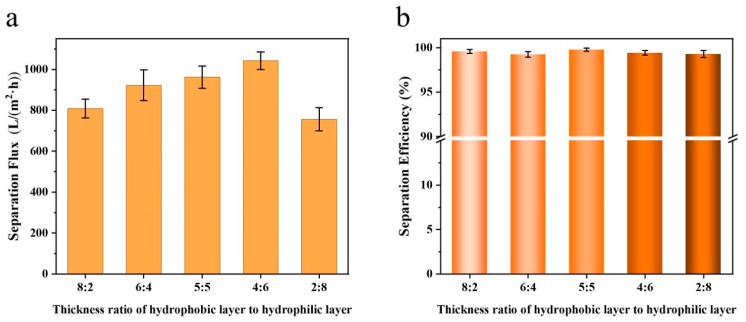
The separation performance of Janus composite nanofiber membranes for water-in-oil emulsion under different thickness ratios of hydrophobic and hydrophilic layers: (**a**) separation flux; (**b**) separation efficiency.

Compared with oil–water mixed systems, the oil and water phases in water-in-oil emulsions are indistinguishable to the naked eye, making them more consistent with practical application scenarios. The DCM-based water-in-water emulsion prepared in this experiment was opaque and white. After filtration through the composite fiber membrane, the filtrate regained transparency. Figure 5 demonstrates the separation performance of Janus composite nanofiber membranes for water-in-oil (DCM) emulsions under different thickness ratios of hydrophobic and hydrophilic layers. As observed in Figure 5a, with the gradual increase in the thickness of the hydrophilic layer, the variation trend of the separation flux of the oil-in-water emulsion was consistent with that of the oil–water mixture, i.e., first increasing and then decreasing. When the thickness ratio of the hydrophobic layer to the hydrophilic layer was 4:6, the separation flux of the fiber membrane for the oil-in-water emulsion reached the maximum value (1042.66 L m^−2^ h^−1^). A comparison between Figure 4a and Figure 5a reveals that under the same thickness ratio, the separation flux of the composite fiber membrane for the water-in-oil emulsion was lower than that for the oil–water mixture. This is because, in water-in-oil emulsions, the large dichloromethane molecules are fragmented into smaller oil droplets under the action of emulsifier, reducing their surface tension. This prevents DCM from aggregating into large droplets, and the gravitational effect of the tiny oil droplets is easily counteracted, thereby slowing down the gravity-driven filtration rate. As shown in Figure 5b, the values for the separation efficiency of the composite fiber membrane for the water-in-oil emulsion were all above 99%. When the thickness ratio was 5:5, the separation efficiency of the fiber membrane was the highest (99.77%). Compared with the separation efficiency for oil–water mixtures, the overall separation efficiency of the composite fiber membrane for water-in-oil emulsions showed a slight improvement.

### 3.4. Comparison of Separation Performance with Other Oil–Water Separation Membranes

The separation performance of the PVDF/SiO_2_-cPVA/PEG Janus composite fiber membrane, which exhibited an optimal performance in water-in-oil emulsion separation, was compared with that of other oil–water separation membranes reported in recent years (Table 2). The results display that under the condition of achieving water-in-oil emulsion separation solely by self-weight, the Janus membrane in this study ranks among the top in both separation flux and separation efficiency.

A comparison with the PAN/FPU/PSF membrane in Reference [43] serves to clarify the focus of this study. Both membranes achieve separation efficiencies in excess of 99.4%, but their design objectives differ. The work in Reference [43] pursued ultra-high flux (1124 L·m^−2^·h^−1^) through constructing a fluorination gradient structure. In contrast, our study demonstrates that regulating the bilayer thickness ratio (optimally 4:6) synergistically optimizes separation performance and mechanical properties. Although the flux of the membrane developed herein (1043 L·m^−2^·h^−1^) is slightly lower, this performance is attained alongside high tensile strength (30.92 MPa) and elongation at break (55.63%), thus providing a balanced performance strategy tailored for applications prioritizing durability. In summary, the comparative results demonstrate the superior performance of the proposed Janus membrane. Despite comparable key parameters to Reference [43], specific differences arise from a distinct design philosophy that balances separation efficiency, flux, and mechanical strength via thickness ratio regulation.

**Table 2:** Comparison of separation performance between this study and other fiber membranes for gravity-driven oil–water separation.

Sample	Separation Flux (L m^−2^ h^−1^)	Separation Efficiency (%)	Reference
PVDF/ZnO	829	-	[44]
HBPU/F-SiO_2_	549	>99	[45]
SiO_2_/zein/PAN	620	98.3	[46]
PAN/PVDF@PVDF-MTES	684	99.5	[33]
PAN/FPU/PSF	1124	99.4	[43]
PVDF/SiO_2_-cPVA/PEG	1043	99.4	This study

### 3.5. The Surface Wetting Performance

**Figure 6:**
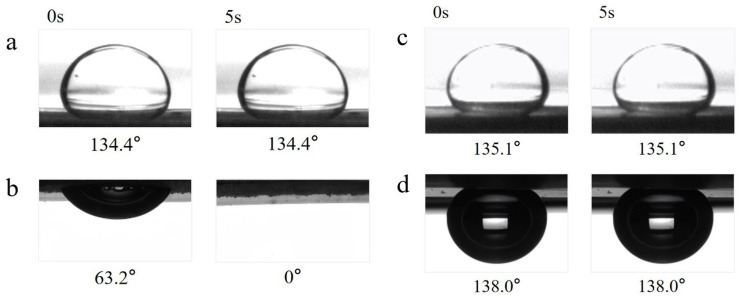
Time-dependent contact angles of nanofiber membrane: (**a**) underoil water contact angle and (**b**) underwater oil contact angle of the hydrophobic side; (**c**) underoil water contact angle and (**d**) underwater oil contact angle of the hydrophilic side.

To further investigate the wetting behavior of the Janus fiber membrane during the oil–water separation process, supplementary tests for the underoil water contact angle and the underwater oil contact angle were conducted (Figure 6). As shown in Figure 6a, the hydrophobic side exhibited underoil hydrophobicity, with an underoil water contact angle of 134.4°, and this contact angle remained unchanged over time. This phenomenon is partly attributed to the intrinsic hydrophobicity of the material itself. On the other hand, a large amount of oil seeping into the pore structure of the hydrophobic-side fiber membrane further hindered water penetration to some extent. When the oil droplet initially touched the surface of the hydrophobic-side fiber membrane underwater, the underwater oil contact angle was 63.2°, which then decreased to 0° within 5 s (Figure 6b). It can thus be inferred that the hydrophobic-side fiber membrane was an underwater oleophilic membrane. This is most likely because the low surface energy of the hydrophobic side enables the fiber membrane to adsorb oil droplets in water.

The hydrophilic side of the nanofiber membrane showed underoil hydrophobicity, with an underoil water contact angle of 135.1°, and this angle did not change over time (Figure 6c). Meanwhile, as displayed in Figure 6d, when the oil drops just touched the surface of the hydrophilic side of the fiber membrane underwater, the underwater oil contact angle was 138°, and this angle did not change with time. It can thus be inferred that the hydrophilic side of the fiber membrane possessed good underwater oleophobicity. This is very likely because when the oil droplet attempts to wet the hydrophilic-side surface, it was repelled by the water on the hydrophilic side surface, resulting in the oil droplet being unable to enter. To sum up, the hydrophobic side of the Janus composite nanofiber membrane was hydrophobic and lipophilic in air, hydrophobic in oil, and oleophilic in water. At the same time, the hydrophilic side of the Janus composite nanofiber membrane was hydrophilic and oleophilic in air, hydrophobic in oil, and oleophobic in water.

### 3.6. Separation Cycle Performance

**Figure 7:**
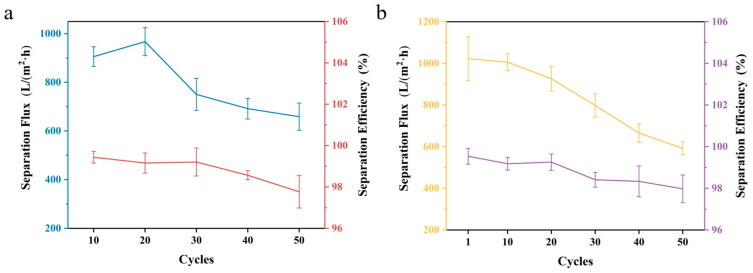
The separation cycle performance of Janus composite nanofiber membranes for (**a**) water-in-DCM and (**b**) water-in-n-hexane.

The separation cycle performance, that is, the variation in the separation performance of the Janus fiber membrane after multiple repeated separation experiments, is of great guiding significance for subsequent practical applications. Figure 7 demonstrates the separation cycle performance of the Janus composite nanofiber membrane for water-in-oil emulsions. With an increase in the number of cycles, the separation flux of the Janus fiber membrane for the water-in-DCM emulsions showed a trend of first increasing and then continuously decreasing (Figure 7a). When the number of cycles reached 20, the separation flux of the fiber membrane increased slightly (from 905.02 to 967.40 L m^−2^ h^−1^). As the number of cycles further increased, the separation flux of the fiber membrane gradually decreased; when the number of cycles reached 50, the separation flux decreased to 658.64 L m^−2^ h^−1^. With the increase in the number of cycles, the separation efficiency of the Janus fiber membrane for the water-in-DCM emulsions generally exhibited a downward trend, but the overall separation efficiency was all above 97.7%. In conclusion, after 50 cycles, the separation performance of the fiber membrane for the water-in-DCM emulsions remained relatively stable.

As clearly shown in Figure 7b, with the continuous increase in the number of cycles, the separation flux of the Janus fiber membrane for water-in-n-hexane emulsions displayed a continuous decreasing trend. The maximum separation flux was 1022.64 L m^−2^ h^−1^ (first cycle), and the minimum was 591.55 L m^−2^ h^−1^ (50th cycle). This is attributed to the fact that the oil blocked some of the through holes of the fiber membrane during the cyclic process. The values for the separation efficiency of the Janus fiber membranes for water-in-n-hexane emulsions were all above 97.9%. In summary, after 50 cycles, the separation performance of the fiber membrane for the water-in-n-hexane emulsion remains relatively stable.

### 3.7. Flexibility Performance

**Figure 8:**
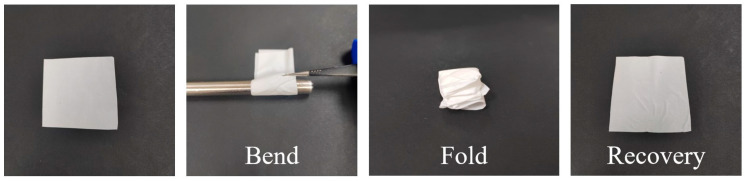
The flexibility performance of Janus composite nanofiber membranes.

The flexibility of fiber membranes is an extremely important indicator for their subsequent practical applications. Figure 8 elaborately presents the flexibility performance of Janus composite nanofiber membranes. After being curled and folded, the Janus composite nanofiber membrane could be unfolded, with its morphology remaining basically unchanged and no fractures or irreparable damage observed. This sufficiently proves that the composite fiber membrane has excellent bending and folding resistance, which can also be confirmed by the outstanding mechanical properties of the fiber membrane.

### 3.8. Collection of Floating Oil in Water

**Figure 9:**
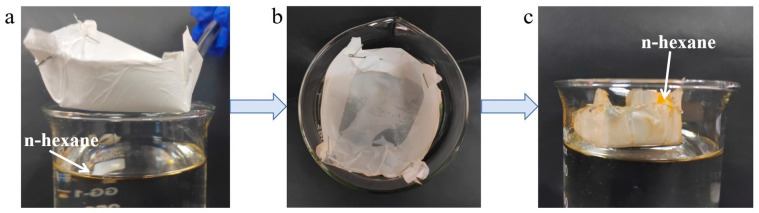
The collection process of floating oil on water by Janus composite nanofiber membranes: (**a**) boat-shaped collector placed at the oil-water interface; (**b**) selective absorption of the oil phase; (**c**) collector fully saturated with oil, showing a color change.

In practical application scenarios, apart from the separation requirements of water-in-oil emulsions, the collection of floating oil on the water surface is also crucial. The ability to collect floating oil on water against gravity is an important means to effectively conserve resources and improve efficiency [47]. Figure 9 illustrates the process of floating oil collection on water by Janus composite nanofiber membranes. First, n-hexane stained with Sudan II was added to a measuring cup containing deionized water, and the composite fiber membrane was shaped into a boat-like form to collect the floating oil on water (Figure 9a). At this point, the hydrophobic side was faced downward to prevent the deionized water from seeping into the fiber membrane. Secondly, the collector was placed on the water surface (Figure 9b), and it can be observed that part of the oil had been absorbed by the fiber membrane, while the membrane remained unwetted by water. After a certain period of time (Figure 9c), the fiber membrane collector was completely soaked in oil, and the color changed from the original white to orange-red, indicating that the fiber membrane successfully collected the floating oil on water without any leakage. This is attributed to the asymmetric wettability of the Janus composite nanofiber membrane, whose forward pressure-bearing capacity is lower than its reverse pressure-bearing capacity, enabling unidirectional transport of oil within the membrane under pressure.

## 4. Conclusions

PVDF/PVA-based Janus fiber membranes with asymmetric wettability were fabricated by the layer-by-layer electrospinning. By comparing mechanical properties and oil–water separation performance, it was determined that the Janus membranes exhibited optimal comprehensive performance when the thickness ratio of the hydrophobic layer to the hydrophilic layer was 4:6. Under these conditions, the fiber membrane achieved a fracture stress of 30.92 ± 2.99 MPa and a fracture strain of 55.63 ± 4.77%. The separation fluxes for the oil–water mixture and the water-in-oil emulsion reached 1888.22 and 1042.66 L m^−2^ h^−1^, respectively, with both the separation efficiencies exceeding 99%. The hydrophobic side of the Janus composite nanofiber membrane was hydrophobic in oil and oleophilic in water, while the hydrophilic side was hydrophobic in oil and oleophobic in oil. After 50 separation cycles with two different water-in-oil emulsions, the Janus fiber membrane maintained stable separation flux and efficiency. Additionally, the fiber membrane possessed excellent bending and folding resistance and a certain capacity for collecting floating oil on water. These characteristics highlight its broad application prospects in oil–water separation, floating oil collection, and related fields. For further development, future work will focus on two interconnected aspects: first, conducting in-depth structural characterization of the Janus membranes and fibers (e.g., via scanning electron microscopy and atomic force microscopy) to elucidate the intrinsic relationship between microstructure, surface properties, and separation performance; second, evaluating the membrane’s separation performance in complex real wastewater systems (e.g., those containing surfactants, inorganic salts, and organic pollutants), which is critical for validating its practical application potential in industrial oily wastewater treatment.

## Figures and Tables

**Table 1:** The breaking stress and strain of Janus composite nanofiber membranes with different thickness ratios of hydrophobic and hydrophilic layers.

Thickness Ratios of Hydrophobic and Hydrophilic Layers	Breaking Stress (MPa)	Breaking Strain (%)
8:2	20.95 ± 2.62	42.48 ± 4.86
6:4	21.02 ± 4.58	61.81 ± 3.77
5:5	32.70 ± 5.26	48.85 ± 9.27
4:6	30.92 ± 2.99	55.63 ± 4.77
2:8	30.02 ± 2.58	70.35 ± 6.08

## Data Availability

The original contributions presented in this study are included in the article. Further inquiries can be directed to the corresponding author.
